# Estimating the Optimal COVID-19 Booster Timing Using Surrogate Correlates of Protection: A Longitudinal Antibody Study in Naïve and Previously Infected Individuals

**DOI:** 10.3390/pathogens14111138

**Published:** 2025-11-10

**Authors:** Yoshihiro Fujiya, Ryo Kobayashi, Makito Tanaka, Ema Suzuki, Shiro Hinotsu, Mami Nakae, Yuki Sato, Yuki Katayama, Masachika Saeki, Yuki Yakuwa, Shinya Nirasawa, Akemi Endoh, Koji Kuronuma, Satoshi Takahashi

**Affiliations:** 1 Department of Infection Control and Laboratory Medicine, Sapporo Medical University School of Medicine, Sapporo 060-8556, Japan; mkt-tnk@sapmed.ac.jp (M.T.); maema@sapmed.ac.jp (E.S.); sato.yuuki@sapmed.ac.jp (Y.S.); y.katayama@sapmed.ac.jp (Y.K.); saeki@sapmed.ac.jp (M.S.); stakahas@sapmed.ac.jp (S.T.); 2 Division of Laboratory Medicine, Sapporo Medical University Hospital, Sapporo 060-8543, Japan; r.kobayashi@sapmed.ac.jp (R.K.); yakuwayu@sapmed.ac.jp (Y.Y.); nirasawa@sapmed.ac.jp (S.N.); endoh@sapmed.ac.jp (A.E.); 3 Department of Biostatistics and Data Management, Sapporo Medical University School of Medicine, Sapporo 060-8556, Japan; hinotsus@sapmed.ac.jp; 4 Division of Infection Control, Sapporo Medical University Hospital, Sapporo 060-8543, Japan; nmami@sapmed.ac.jp (M.N.); kuronumak@sapmed.ac.jp (K.K.); 5 Department of Respiratory Medicine and Allergology, Sapporo Medical University School of Medicine, Sapporo 060-8556, Japan

**Keywords:** COVID-19, mRNA vaccine, booster timing, antibody titer, mixed-effects model, correlate of protection, surrogate virus neutralization test

## Abstract

Standardized, one-size-fits-all COVID-19 booster schedules may be suboptimal due to individual variation in immune backgrounds, particularly prior infection, which induces robust hybrid immunity. This study estimated optimal booster timing by modeling antibody decay in relation to surrogate correlates of protection (CoP). In a prospective cohort of 177 Japanese healthcare workers, we longitudinally monitored anti-spike receptor-binding domain (S-RBD) antibody titers following BNT162b2 vaccination. Participants were stratified into SARS-CoV-2-naïve and previously infected groups. Mixed-effects models were developed to predict when antibody titers would decline below predefined CoP thresholds. The model estimated optimal booster timing after a two-dose primary series to be 3–5 months for naïve individuals and approximately one year for those with prior infection. Following a third dose, the estimated interval extended to 8–12 months for the naïve group and 1.5–2 years for the previously infected group. These substantial differences underscore the limitations of uniform booster schedules. Our findings provide a quantitative framework for personalized vaccination strategies based on individual antibody profiles and immune status, thereby optimizing protection.

## 1. Introduction

The coronavirus disease 2019 (COVID-19) pandemic has triggered an unprecedented global public health crisis. In response, the rapid development and widespread administration of mRNA vaccines targeting the SARS-CoV-2 spike protein have proven remarkably effective, particularly in reducing severe disease and mortality [1,2,3]. Vaccine-induced humoral immunity, especially neutralizing antibody titers, has been shown to correlate with protection against symptomatic infection [4]. However, early reports indicated that these antibody titers wane over time [5,6]. This decline, combined with the emergence of viral variants exhibiting immune escape properties, has reduced protective efficacy [4] and led to breakthrough infections [7,8]. Consequently, the importance of booster vaccination to sustain long-term protection has become widely recognized.

This study aimed to estimate the optimal COVID-19 booster timing based on anti-SARS-CoV-2 antibody decay modeling and predefined surrogate correlates of protection (CoP), defined as IgG titers corresponding to high neutralizing activity.

Many countries initially adopted uniform booster schedules; however, vaccination strategies have since shifted toward annual updates, particularly for populations at high risk of severe disease [9,10]. Nevertheless, such populations are heterogeneous, and evidence supporting personalized booster timing that considers infection history and immune status remains limited. With the growing prevalence of hybrid immunity—resulting from both vaccination and prior infection—immune durability has proven stronger than with vaccination alone [11]. Conversely, immunocompromised individuals show variable antibody decay and may require more frequent boosters [12,13]. These observations indicate that a uniform, “one-size-fits-all” booster policy may be inadequate. Modeling antibody decay kinetics based on correlates of protection (CoP) could provide a framework for individualized, evidence-based vaccination strategies [14,15].

In a prospective cohort of Japanese healthcare workers, we longitudinally monitored anti-spike receptor-binding domain (S-RBD) antibody titers after mRNA vaccination in both SARS-CoV-2-naïve and previously infected individuals. Using these decay curves, we developed a regression model to estimate the optimal booster timing based on CoP [16]. In this study, the CoPs were used as an antibody threshold indicating when a booster vaccination should be considered. Although the emergence of the Omicron variant shifted the vaccination goal toward preventing severe disease, previous studies have shown that antibody titers remain predictive of protection against infection and booster efficacy for earlier variants [17]. This study, therefore, focuses on pre-Omicron protection, aiming to provide a framework for evaluating the efficacy and timing of future mRNA booster vaccinations.

## 2. Materials and Methods

### 2.1. Study Design and Participants

We conducted an observational longitudinal study without any intervention from March 2021 to June 2022. Participants were healthcare workers affiliated with Sapporo Medical University Hospital, a tertiary referral hospital providing medical care for patients with COVID-19. They were recruited through an internal announcement before the vaccination program began. All participants received COVID-19 vaccination as part of the national immunization program, independent of the study protocol. This was not an intervention study. They were 20 years of age or older and represented various professions, including doctors, nurses, pharmacists, clinical engineers, clinical laboratory technicians, radiology technicians, and medical clerks. Written informed consent was obtained from all participants, and the study was approved by the local ethics committee of Sapporo Medical University (approval number: 2-1-87).

**Figure 1:**
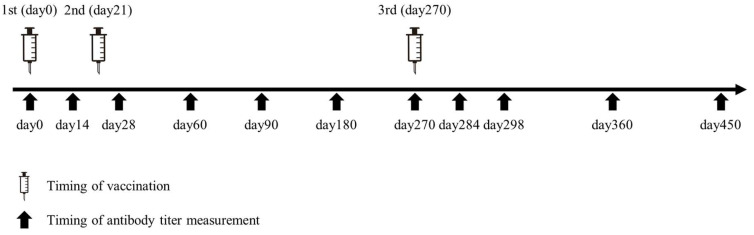
Timeline of BNT162b2 vaccine administration and antibody titer measurements in this study.

In accordance with the recommendation by the Japanese Ministry of Health, Labour and Welfare, participants received two doses of a monovalent (ancestral) mRNA COVID-19 vaccine manufactured by Pfizer/BioNTech (BNT162b2), administered 21 days apart from March to April 2021. A booster dose (third dose) was subsequently administered with an 8-month interval, between December 2021 and January 2022 (Figure 1).

Participants were divided into three groups based on their COVID-19 history prior to the first vaccination: (1) naïve group with no history of infection, (2) prior-infected group 1 (infected within 6 months), and (3) prior-infected group 2 (infected more than 6 months earlier). Regarding the determination of prior SARS-CoV-2 infection before vaccination, individuals were classified as previously infected if they had either a PCR-confirmed SARS-CoV-2 infection or were seropositive for anti-nucleocapsid IgG (ARCHITECT SARS-CoV-2 IgG; Abbott, Chicago, IL, USA) prior to enrollment. Participants who contracted COVID-19 during the study period were excluded from the analyses after infection. In addition, individuals who did not receive all three scheduled vaccine doses, as well as those classified as non-responders, defined as those whose antibody titers did not increase following vaccination compared with pre-vaccination levels, were also excluded. Participants who missed one or more blood testing were not excluded from the study; analyses were based on available data.

**Figure 2:**
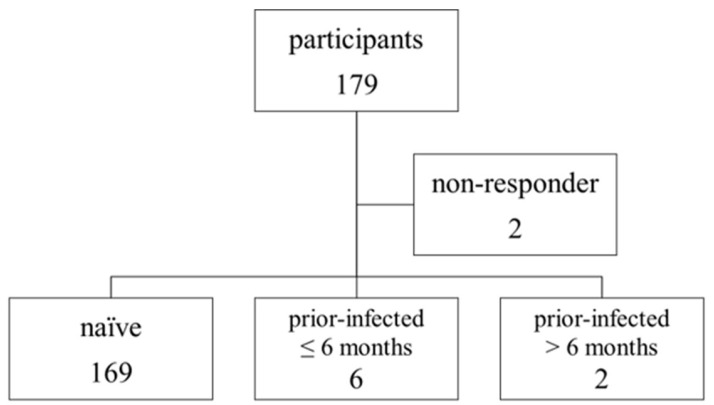
Grouping of study participants based on COVID-19 history. Two participants were non-responders to the vaccine and were excluded from the analysis.

A total of 179 participants were enrolled in this study. Two participants were excluded from the analysis because they were non-responders to the vaccine, resulting in 177 participants being included in the final analysis (Figure 2). Of these, six had been infected with COVID-19 within 6 months prior to initial vaccination (prior-infected group 1), and two had been infected more than 6 months earlier (prior-infected group 2).

### 2.2. Data and Sample Collection

Information on participants’ backgrounds was obtained through a questionnaire. Blood samples were collected at eleven time points during the study period (Figure 1): before the first vaccination (day 0), and at 2 weeks (day 14), 4 weeks (day 28), 2 months (day 60), 3 months (day 90), 6 months (day 180), and 9 months (day 270) after the first vaccination; followed by 2 weeks (day 284), 4 weeks (day 298), 3 months (day 360), and 6 months (day 450) after the third vaccination. At day 270, samples were collected prior to the third vaccination.

### 2.3. Measurement of Anti-SARS-CoV-2 Antibody

Anti-SARS-CoV-2 antibodies were measured using two types of reagents, in accordance with the manufacturer’s instructions. Anti-nucleocapsid IgG (N-IgG) was measured at day 0 using the ARCHITECT SARS-CoV-2 IgG assay (Abbott, Chicago, IL, USA) to assess prior exposure to SARS-CoV-2. The results were reported as a cutoff index (S/C; signal sample/cutoff) and interpreted qualitatively. An S/C ≥ 1.40 was considered positive. The S-RBD antibodies were measured using the SARS-CoV-2 IgG II Quant assay (Abbott, Chicago, IL, USA) to evaluate antibody titers following vaccination (S-IgG). The results were reported quantitatively in arbitrary units per milliliter (AU/mL). Titers ≥ 50 AU/mL were considered positive. Participants whose antibody titers did not exceed 50 AU/mL even after receiving the second dose were classified as non-responders. Values above the upper measurement limit (40,000 AU/mL) were quantified following dilution, as recommended by the manufacturer. All measurements were performed immediately after blood collection. Both assays were run on the ARCHITECT i2000SR analyzer (Abbott, Chicago, IL, USA).

### 2.4. Surrogate Indicator for Correlates of Protection

Khoury et al. and Cromer et al. have shown that neutralizing antibody titers correlate with protection against symptomatic infection [4,17]. We evaluated the neutralizing capacity against SARS-CoV-2 to estimate the CoP. Tan et al. reported 84% inhibition in a surrogate virus neutralization test (sVNT) in COVID-19 patients 14–61 days after disease onset [18]. Our research team previously demonstrated that an S-IgG level corresponding to an 84% inhibition rate was 2976 AU/mL in the ARCHITECT SARS-CoV-2 IgG II assay [16]. Additionally, Pezzati et al. reported that an S-IgG cut-off level of 1236.1 AU/mL using the same assay was associated with high neutralizing activity (>56% inhibition) with excellent sensitivity and specificity [19]. Therefore, we used 2976 AU/mL and 1236.1 AU/mL as surrogate indicators for CoP for the mRNA COVID-19 vaccine in this study.

### 2.5. Statistical Analyses

As mentioned above, participants missing any antibody titer at a given time point were retained; analyses were based on available data without imputing missing values. Statistical analyses were performed using JMP Student Edition 18 statistical software (SAS Institute, Cary, NC, USA). Levels of S-IgG at each measurement point were expressed as geometric mean titers (GMTs) with 95% confidence intervals, calculated using log-transformed data. GMTs between groups were compared using the Mann–Whitney U test or Kruskal–Wallis test. A two-tailed *p*-value of <0.05 was considered statistically significant. We conducted a regression analysis of the relationship between the number of days after vaccination and S-IgG titers to estimate the optimal timing for the next vaccination. Based on previous studies demonstrating exponential decay of antibody titers over time [20], we used a mixed-effects model to analyze the relationship between the base-10 logarithm of S-IgG titers and the number of days post-vaccination.

## 3. Results

### 3.1. Characteristics of Participants

As noted above, a total of 177 participants were included in the final analysis: 169 in the naïve group, 6 in prior-infected group 1, and 2 in prior-infected group 2 (Figure 2). Baseline characteristics of the analyzed participants are presented in Table 1. The median age was 41 years (range: 23–64), with the majority in their 30s and 40s; 98 (55%) were male. Among all participants, 114 (59%) were doctors or nurses, and 111 (63%) had been involved in the treatment of patients with COVID-19. Among the eight participants with a history of prior infection, six (75%) were doctors or nurses, and seven (88%) were directly involved in the treatment of COVID-19 patients. Although some participants had comorbidities, few were immunocompromised. Six participants contracted COVID-19 after receiving the third vaccine dose.

### 3.2. Dynamics of Vaccine Response Among Naïve and Prior-Infected Group

**Figure 3:**
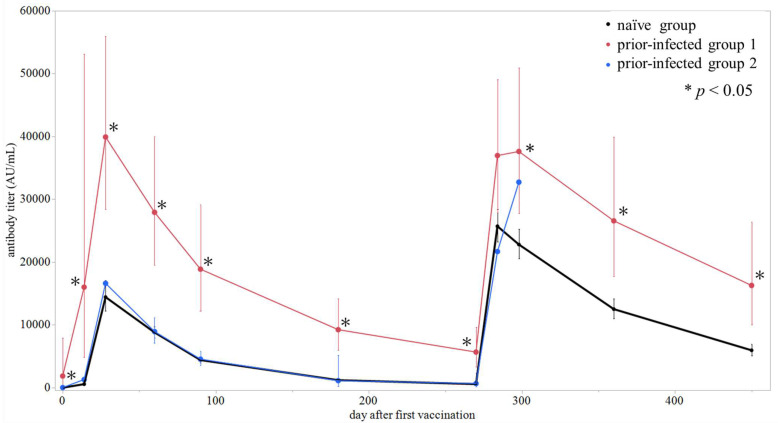
Dynamics of SARS-CoV-2 anti-spike receptor-binding domain (S-RBD) antibody titers after mRNA vaccine BNT162b2. Dots represent the geometric mean titers (GMTs) for each group at each measurement point, and vertical lines represent 95% confidence intervals. Black: naïve group; red: prior-infected group 1; blue: prior-infected group 2. * The GMT in the indicated group is significantly higher than that in the naïve group (Mann–Whitney U test, *p* < 0.05).

We evaluated the kinetics of S-RBD antibody titers following mRNA COVID-19 vaccination in each group. The GMTs at each measurement point and their chronological changes for each group are presented in Table 2 and Figure 3, respectively. Due to the small sample size (n = 2), results for prior-infected group 2 were presented descriptively. Before vaccination (day 0), the GMTs were 1.9 AU/mL in the naïve group and 1857 AU/mL in prior-infected group 1, showing a significant difference (*p* < 0.05), while all participants in prior-infected group 2 were seronegative. After the first dose, antibody titers rose sharply in all groups, with prior-infected group 1 reaching 16,008 AU/mL by day 14—comparable to the naïve group’s peak after the second dose. Throughout the observation period, antibody titers in prior-infected group 1 remained significantly higher than those in the naïve group, except at day 284, whereas titers in the prior-infected group 2 followed a similar trend to those in the naïve group. After the third dose, antibody titers again increased rapidly, followed by gradual decline. Data for prior-infected group 2 after the third dose were unavailable. We also evaluated the kinetics of antibody titers in the naïve group according to sex and age group. The kinetics and GMTs at each measurement point were not significantly different regardless of sex or age group (Figures S1 and S2, Table S1). Incidentally, individual antibody kinetics of six participants who contracted COVID-19 after the third vaccination are shown in Figure S3.

### 3.3. Timing of Booster Vaccine Administration

**Figure 4:**
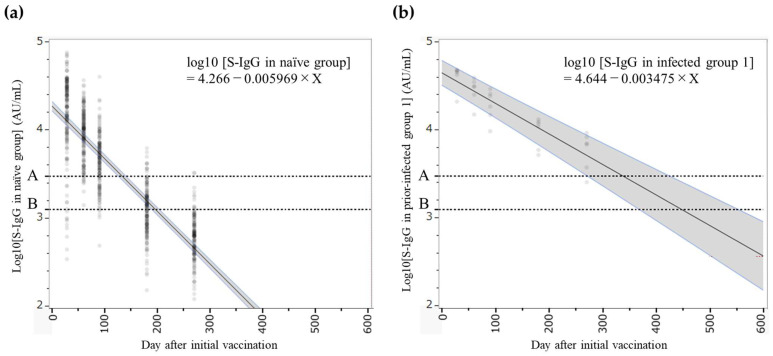
Mixed-effects model of the base-10 logarithm of anti-SARS-CoV-2 spike protein receptor-binding domain (S-IgG) antibody titers after the second dose in the naïve group (**a**) and the prior-infected group 1 (**b**). Dots represent the distribution of S-IgG titers at each measurement point. The gray shaded areas indicate 95% confidence intervals for the regression lines in each group. Horizontal dotted lines indicate the surrogate CoPs used in this study (A; 2976 AU/mL, B; 1236.1 AU/mL).

We conducted regression analyses based on the distribution of antibody titers at each measurement point and applied the surrogate CoPs to evaluate the timing for the next vaccine administration. Due to the small sample size (n = 2), results for prior-infected group 2 were not included in formal modeling. The antibody titer at a given time after the first vaccination was predicted using the following equations (Figure 4):

log10 [S-IgG in the naïve group] = 4.266 − 0.005969 × X

log10 [S-IgG in prior-infected group 1] = 4.644 − 0.003475 × X

Furthermore, Figure S3 illustrates the individual antibody kinetics for the naïve and prior-infected group 1. The interindividual variation in the antibody decay rates within each group was not statistically significant (*p* = 0.27 and *p* = 0.48, respectively).

Based on these models, antibody titers in the naïve group were predicted to reach 2976 AU/mL in 132 days (95% confidence interval [CI]; 119–146) and 1236.1 AU/mL in 196 days (95% CI; 189–204) after the first vaccination, whereas in prior-infected group 1, the predicted times to reach 2976 AU/mL and 1236.1 AU/mL were 337 days (95% CI; 270–426) and 446 days (95% CI; 366–554), respectively. Following a two-dose primary vaccination series administered at a three-week interval, the optimal booster timing was estimated to be approximately 3–5 months after the second dose for the naïve group and approximately one year for prior-infected group 1.

**Figure 5:**
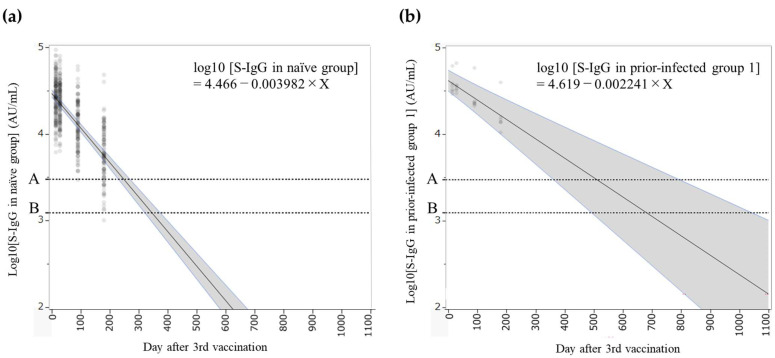
Mixed-effects model of the base-10 logarithm of anti-SARS-CoV-2 spike protein receptor-binding domain (S-IgG) antibody titers after the third dose in the naïve group (**a**) and the prior-infected group 1 (**b**). Dots represent the distribution of S-IgG titers at each measurement point. The gray shaded areas indicate 95% confidence intervals for the regression lines in each group. Horizontal dotted lines indicate the surrogate CoPs used in this study (A; 2976 AU/mL, B; 1236.1 AU/mL).

Similarly, the antibody titer at a given time after the third vaccination (booster dose) was predicted using the following equations (Figure 5):

log10 [S-IgG in the naïve group] = 4.466 − 0.003982 × X

log10 [S-IgG in prior-infected group 1] = 4.619 − 0.002241 × X

These models predicted that antibody titers in the naïve group would reach 2976 AU/mL in 249 days (95% CI; 230–271) and 1236.1 AU/mL in 345 days (95% CI; 320–375) after the booster dose, while in prior-infected group 1, these thresholds would be reached in 511 days (95% CI; 362–796) and 681 days (95% CI; 493–1045), respectively. In the naïve group, the slope of the mixed-effects model considering individual heterogeneity remained statistically significant (*p* < 0.01); however, the slope for prior-infected group 1 was not statistically significant (*p* = 0.17). Accordingly, if an additional booster dose is to be administered, the timing is suggested to be 8–12 months after the third dose for the naïve group. For reference, although the model for prior-infected group 1 was not sufficiently robust, the approximate timing for an additional booster in this group was estimated to be around 1.5–2 years.

## 4. Discussion

This study aimed to model the decay kinetics of S-RBD antibody titers after mRNA vaccination in a cohort of Japanese healthcare workers and to estimate the optimal timing for booster vaccination based on individual SARS-CoV-2 infection history. While neutralizing antibody titers have been shown to serve as a CoP against symptomatic infection [4,17], few studies have applied this concept to estimate specific booster timings tailored to individual immune backgrounds. Therefore, this study represents an important step toward translating the CoP concept into clinical application and advancing personalized vaccination strategies.

The surrogate CoP thresholds used in this study were based on antibody titers previously shown by our group and Pezzati et al. to correlate with strong neutralizing activity in an sVNT [16,19]. Although the conventional virus neutralization test (cVNT) remains the gold standard, its technical complexity and requirement for BSL-3 facilities limit clinical use. In contrast, the sVNT can be safely and efficiently performed in BSL-2 settings and shows strong correlation with both cVNT and S-IgG titers [16,18,19], supporting the use of S-IgG as a practical surrogate for neutralizing capacity. Two CoP thresholds were applied, reflecting reported ranges in previous studies [21,22], to express optimal booster timing as a clinically meaningful range rather than a single estimate.

Our dataset consists of repeated antibody measurements from the same participants. Therefore, we analyzed the data using a mixed-effects model, treating days as a fixed effect and individual participants as a random effect, thereby accounting for within-subject correlation. With regard to missing data, the proportion after the second dose was low (3.8%), which was considered to be missing at random. Although the model fit after the third dose was suboptimal in prior-infected group 1, we present the estimates with confidence intervals and describe them as exploratory.

According to our model, for the purpose of preventing symptomatic infection with the ancestral strain, the optimal timing for a booster dose after the primary two-dose series was estimated to be approximately 3–5 months for the naïve group and approximately one year for prior-infected group 1. At the time this study was conducted, many countries recommended a uniform booster schedule approximately 8 months after the second dose. Our estimate of 3–5 months for the naïve group suggests that a shorter interval than the uniform schedule in place at the time may have been more appropriate to maintain protective antibody levels—a finding consistent with several prior studies reporting faster-than-expected antibody decay [5,23]. Conversely, the one-year estimate for prior-infected group 1 suggests that vaccination at the standard interval may have been unnecessary, considering the robust durability of hybrid immunity. These findings reinforce the need for a personalized approach to booster scheduling.

Furthermore, our model suggested longer intervals for the subsequent booster following the third dose: approximately one year for the naïve group and 1.5–2 years for prior-infected group 1. This finding suggests that immune memory likely matured after the third dose, resulting in more gradual antibody decay compared to that observed after the second dose. Similar antibody kinetics have been reported in other studies [20]. However, it is important to note that these long-term booster interval estimates are theoretical values based on ideal conditions—specifically, the assumption of a continuously circulating virus without antigenic variation. In real-world pandemic settings, the emergence of new variants with immune escape properties becomes a critical factor in determining vaccination schedules. Nevertheless, the theoretical estimate of approximately one year for the naïve group aligns conceptually with the subsequent shift toward an annual vaccination strategy, similar to that used for seasonal influenza. Among healthy individuals with hybrid immunity in the prior-infected group 1, although the model fit was suboptimal, a longer interval between doses may be adequate, and a personalized strategy could help prevent unnecessary booster administration.

Recent evidence also supports the sustained immunological benefits of repeated booster vaccination. Speletas et al. demonstrated that both IgG and IgA antibody responses significantly increased following successive booster doses, contributing to a markedly reduced risk of fatal COVID-19 outcomes [24]. These findings provide real-world immunological validation for our model-based prediction that booster vaccination contributes not only to maintaining antibody levels but also to enhancing durable protection against severe disease.

The strong priming effect of hybrid immunity was evident in this study. The prior-infected group 1 reached a GMT of 16,008 AU/mL only 14 days after the first dose—a level comparable to the naïve group’s peak after the second dose—indicating that a single vaccine dose can elicit a potent boost in previously infected individuals [25,26]. In contrast, the comparable peak titers observed after the second and third doses suggest a ceiling effect in antibody production. Consistent with previous reports [27,28], additional boosting at short intervals provided little incremental benefit, likely due to saturation of memory B-cell stimulation. This pattern implies limited utility of uniform booster schedules for individuals with robust immune memory.

Interestingly, prior-infected group 2, infected more than six months before vaccination, showed antibody kinetics similar to those of the naïve group. Previous studies suggest that longer infection-to-vaccination intervals enhance immune quality by promoting memory B-cell maturation and broader neutralizing activity [29]. However, in our study, the short-term antibody boost after the first dose was weaker in group 2 than in group 1, consistent with the findings of Buckner et al. [30]. These results suggest that while extended intervals may improve immune quality, the immediate priming effect on antibody production may diminish over time. Given the very small sample size of prior-infected group 2, this interpretation remains preliminary and requires further investigation.

The findings of this study reinforce that a “one-size-fits-all” approach to vaccination—characterized by a uniform interval for all individuals—is suboptimal, particularly in modern populations with diverse immunological backgrounds. Such standardization may result in unnecessary or excessive vaccination, especially among individuals with hybrid immunity, thereby leading to inefficient use of public health resources. Although this study provides a proof of concept for estimating individualized booster timing based on antibody kinetics and correlates of protection, it is not intended to recommend routine antibody testing at this stage. Nevertheless, incorporating infection history and antibody measurements as supplementary tools may support booster scheduling decisions, particularly among high-risk populations. Asymptomatic infection and immunocompromised individuals remain important areas for future investigation.

This study has several limitations. First, it was conducted at a single center, and the number of participants in the prior-infected groups was small, limiting the statistical power to detect subtle differences. Especially, the fitness of our model after the third dose in prior-infected group 1 showed limited, and the confidence intervals were wide due to the small sample size. Accordingly, the estimates derived from this model are presented for reference only and should be interpreted with caution. Second, the study population consisted of relatively young and healthy healthcare workers; thus, the findings may not be generalizable to other populations, such as older adults or immunocompromised individuals. Third, the antibody data and CoP used in this analysis were based on the ancestral SARS-CoV-2 strain. The current SARS-CoV-2 variants with immune-escape would alter the generalizability of our findings based on the ancestral strain. Fourth, the CoP was derived from surrogate markers and not from direct clinical outcome assessments. Finally, this study focused exclusively on humoral immune responses and did not evaluate cellular immunity, which also plays a critical role in protection against SARS-CoV-2 [31]. T cell-mediated immunity contributes substantially to long-term protection and mitigation of disease severity, even after humoral antibody levels wane. Booster vaccination has also been shown to induce cellular immune responses [31]. Future research should incorporate T-cell-based analyses to further refine the assessment of protective immunity in vaccine evaluation strategies.

## 5. Conclusions

The antibody decay model following SARS-CoV-2 vaccination differed significantly based on prior infection history. Our model suggests that, for the purpose of preventing symptomatic infection with the ancestral strain, the appropriate timing for a booster dose after the primary two-dose series may be 3–5 months for naïve individuals and approximately one year for those with prior infection. These findings highlight the limitations of a uniform vaccination schedule and provide a scientific basis for the development of personalized vaccination strategies based on individual antibody profiles and immune backgrounds. However, these estimated time windows should be interpreted with caution as indicative ranges rather than fixed thresholds, given the variability and uncertainty inherent in model-based estimates. Nevertheless, they offer useful insights for optimizing booster schedules for future mRNA vaccines.

## Figures and Tables

**Table 1:** Baseline characteristics of participants included in the analysis (n = 177).

			Total (n = 177)	Naïve (n = 169)	Prior-Infected ≤ 6 Months (n = 6)	Prior-Infected > 6 Months (n = 2)
Characteristics, n (%)						
	Age, years					
		Median (range)	41 (23–64)	41 (23–64)	46 (28–57)	44.5 (43–46)
		20–29	23 (13)	22 (13)	1 (17)	0 (0)
		30–39	58 (33)	57 (34)	1 (17)	0 (0)
		40–49	68 (38)	64 (38)	2 (33)	2 (100)
		50–59	25 (14)	23 (14)	2 (33)	0 (0)
		≥60	3 (2)	3 (2)	0 (0)	0 (0)
	Sex					
		Male	98 (55)	96 (57)	1 (17)	1 (50)
		Female	79 (45)	73 (43)	5 (83)	1 (50)
	Occupation					
		Doctor	58 (33)	57 (34)	0 (0)	1 (50)
		Nurse	46 (26)	41 (24)	5 (83)	0 (0)
		Laboratory technician	44 (25)	43 (25)	0 (0)	1 (50)
		Radiology technician	14 (8)	14 (8)	0 (0)	0 (0)
		Clinical engineer	7 (4)	7 (4)	0 (0)	0 (0)
		Pharmacist	5 (3)	4 (2)	1 (16)	0 (0)
		Others	3 (2)	3 (2)	0 (0)	0 (0)
	Involved in the treatment of COVID-19 patients		111 (63)	104 (62)	6 (100)	1 (50)
Comorbidities, n (%)						
		Allergic rhinitis or hay fever	13 (7)	13 (8)	0 (0)	0 (0)
		Hypertension	7 (4)	7 (4)	0 (0)	0 (0)
		Athma	7 (4)	7 (4)	0 (0)	0 (0)
		Dyslipidemia	4 (2)	4 (2)	0 (0)	0 (0)
		Hyperuricemia	4 (2)	4 (2)	0 (0)	0 (0)
		Chronic kidney disease	2 (1)	2 (1)	0 (0)	0 (0)
		Inflammatory bowel disease	2 (1)	2 (1)	0 (0)	0 (0)
		Cancer	2 (1)	2 (1)	0 (0)	0 (0)
		Others	22 (12)	22 (13)	0 (0)	0 (0)
		Unknown	20 (11)	20 (12)	0 (0)	0 (0)
Infected after vaccination, n (%)			6 (3)	6 (4)	-	-

**Table 2:** Geometric mean titers (GMTs) of anti-SARS-CoV-2 spike protein receptor-binding domain antibodies at each measurement point in each group.

Prior COVID-19 Status	Day 0		Day 14		Day 28		Day 60		Day 90		Day 180		Day 270		Day 284		Day 298		Day 360		Day 450	
	n	GMT (95% CI)	n	GMT (95% CI)	n	GMT (95% CI)	n	GMT (95% CI)	n	GMT (95% CI)	n	GMT (95% CI)	n	GMT (95% CI)	n	GMT (95% CI)	n	GMT (95% CI)	n	GMT (95% CI)	n	GMT (95% CI)
Naïve	169	1.9 (1.7–2.3)	166	563 (490–697)	164	14,424 (12,207–17,044)	162	8800 (7962–9727)	152	4400 (3941–4913)	153	1195 (1077–1327)	139	577 (520–640)	133	25,703 (23,243–28,423)	122	22,799 (20,565–25,277)	102	12,508 (11,020–14,198)	94	5952 (5111–6932)
Prior-infected ≤ 6 months	6	1857 (436–7902)	6	16,008 (4827–53,084)	6	39,915 (28,457–55,987)	6	27,926 (19,482–40,029)	6	18,873 (12,229–29,128)	6	9237 (6017–14,180)	6	5671 (3335–9644)	6	36,963 (27,842–49,074)	6	37,610 (27,775–50,929)	6	26,564 (17,688–39,894)	6	16280 (10,051–26,370)
Prior-infected > 6 months *	2	3.6, 114.6	2	262, 6437	2	12,528, 22,041	2	8781, 9097	2	4455, 4636	2	992, 1263	2	597, 728	2	13,256, 35,454	1	32,729	0	NA	0	NA

NA; Not available. The range in parentheses represents the 95% confidence interval (95% CI). * As only two participants met the eligibility criteria, the raw data for each individual were presented.

## Data Availability

The data presented in this study are available from the corresponding author upon reasonable request. Restrictions apply due to the privacy of study participants.

## Supplementary Materials

The following supporting information can be downloaded at: https://www.mdpi.com/article/10.3390/pathogens14111138/s1, Figure S1: Dynamics of SARS-CoV-2 anti-spike receptor-binding domain antibody titers by sex in the naïve group after BNT162b2 mRNA vaccination; Figure S2: Dynamics of SARS-CoV-2 anti-spike receptor-binding domain antibody titers by age group in the naïve group after BNT162b2 mRNA vaccination; Figure S3: Individual kinetics of SARS-CoV-2 anti-spike receptor-binding domain antibody titers (S-IgG) in naïve group and prior-infected group 1 after initial vaccination; Figure S4: Individual kinetics of SARS-CoV-2 anti-spike receptor-binding domain antibody titers (S-IgG) in naïve group and those who became infected after the 3rd dose (n = 6); Table S1: Geometric mean titers (GMTs) of anti-SARS-CoV-2 spike protein receptor-binding domain antibodies at each measurement point by sex and age group in the naïve group.

## Abbreviations

The following abbreviations are used in this manuscript:

COVID-19	Coronavirus disease 2019
CoP	Correlate of protection
SARS-CoV-2	Severe acute respiratory syndrome coronavirus 2
S-RBD	Anti-spike receptor-binding domain
N-IgG	Anti-nucleocapsid IgG
S-IgG	Anti-spike protein IgG
sVNT	Surrogate virus neutralization test
GMT	Geometric mean titer
cVNT	Conventional virus neutralization test
BSL	Biosafety level
