# Supplementary material for: Estimating the Optimal COVID-19 Booster Timing Using Surrogate Correlates of Protection: A Longitudinal Antibody Study in Naïve and Previously Infected Individuals
Source: Pathogens. 2025 Nov 10;14(11):1138. doi: 10.3390/pathogens14111138 (PMC12655711; doi:10.3390/pathogens14111138)
Supplement: Supplementary file 1 [file pathogens-14-01138-s001.zip › pathogens-3847574-supplementary.pdf]

Supplementary Materials

Figure S1. Dynamics of SARS-CoV-2 anti–spike receptor-binding domain antibody titers by sex in the naïve group after BNT162b2 mRNA vaccination. Dots represent geometric mean titers (GMTs) at each measurement point; vertical lines represent 95% confidence intervals. Red: female; blue: male.

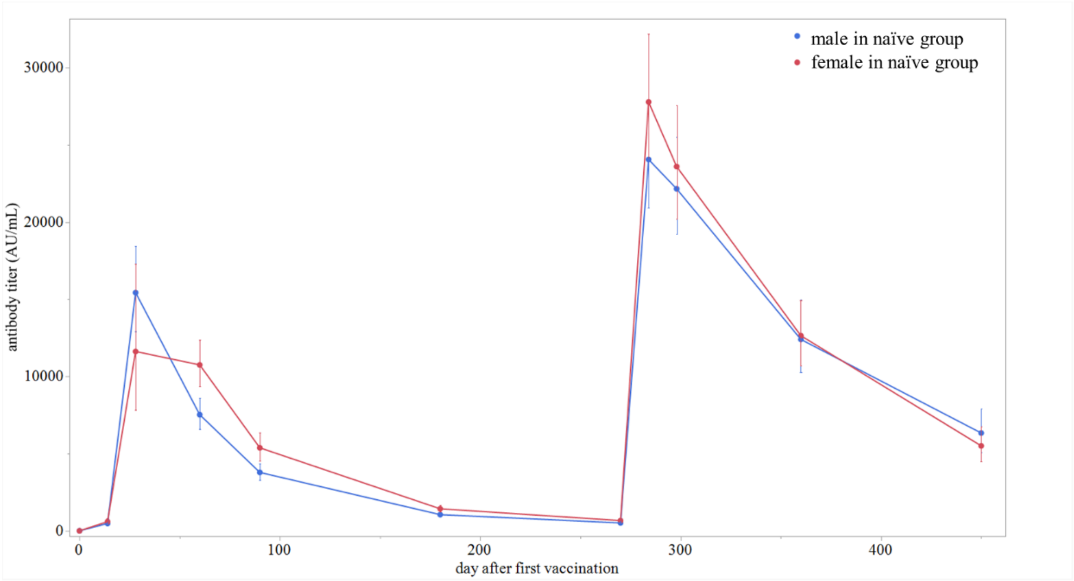

Figure S2. Dynamics of SARS-CoV-2 anti–spike receptor-binding domain antibody titers by age group in the naïve group after BNT162b2 mRNA vaccination. Dots represent geometric mean titers (GMTs) at each measurement point; vertical lines represent 95% confidence intervals. Blue: 20–29 years; red: 30–39 years; green: 40–49 years; purple: 50–59 years; orange: ≥60 years.

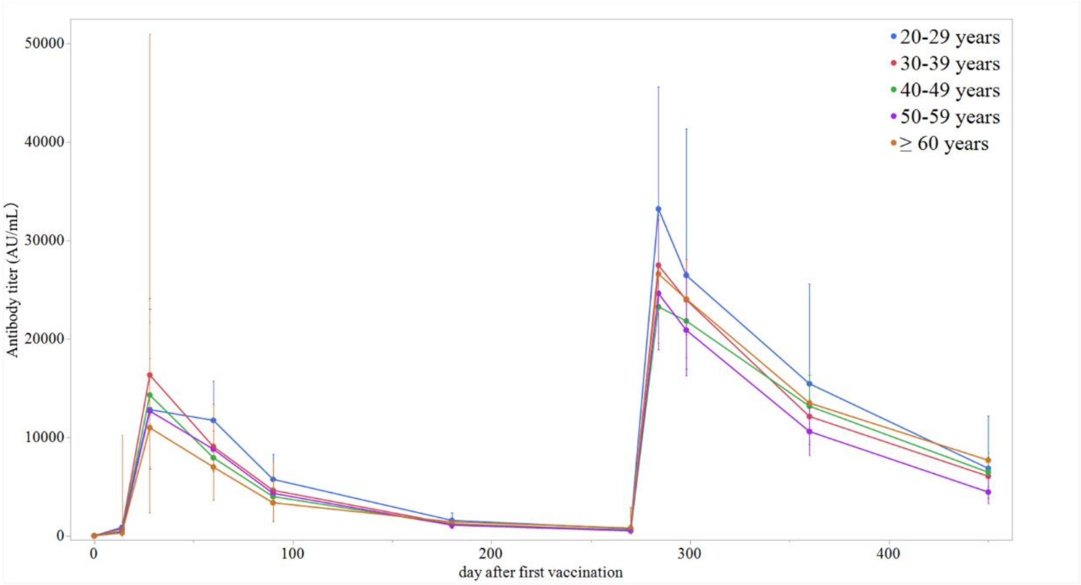

Figure S3. Individual kinetics of SARS-CoV-2 anti–spike receptor-binding domain antibody titers (S-IgG) in naïve group and prior-infected group 1 after initial vaccination. Blue: naïve group; red: prior-infected group 1.

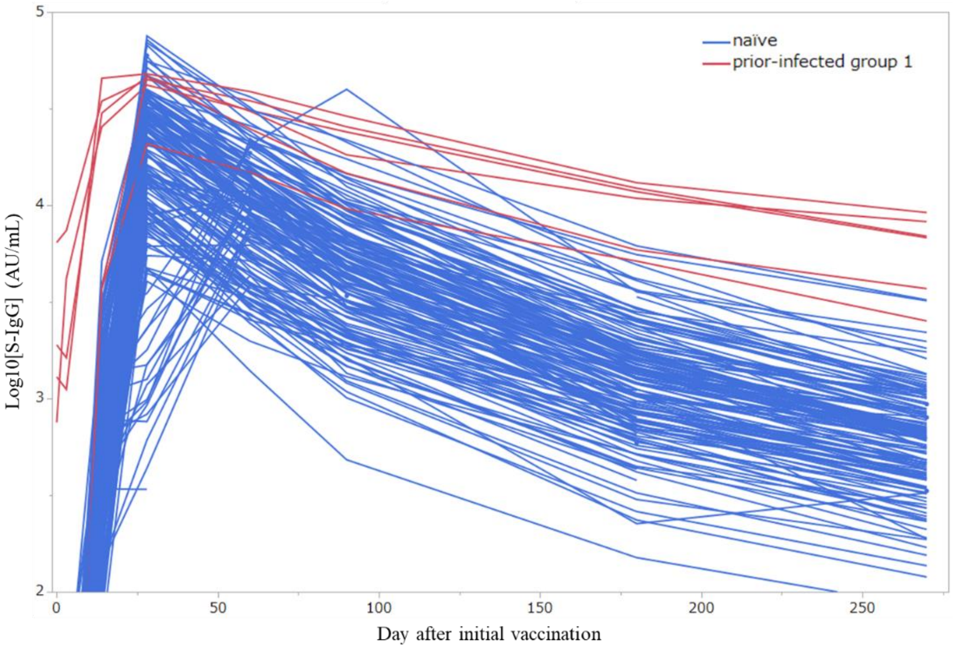

Figure S4. Individual kinetics of SARS-CoV-2 anti–spike receptor-binding domain antibody titers (S-IgG) in naïve group and those who became infected after the 3rd dose (n=6). Light blue: naïve group; orange: those who became infected after the third vaccine dose.

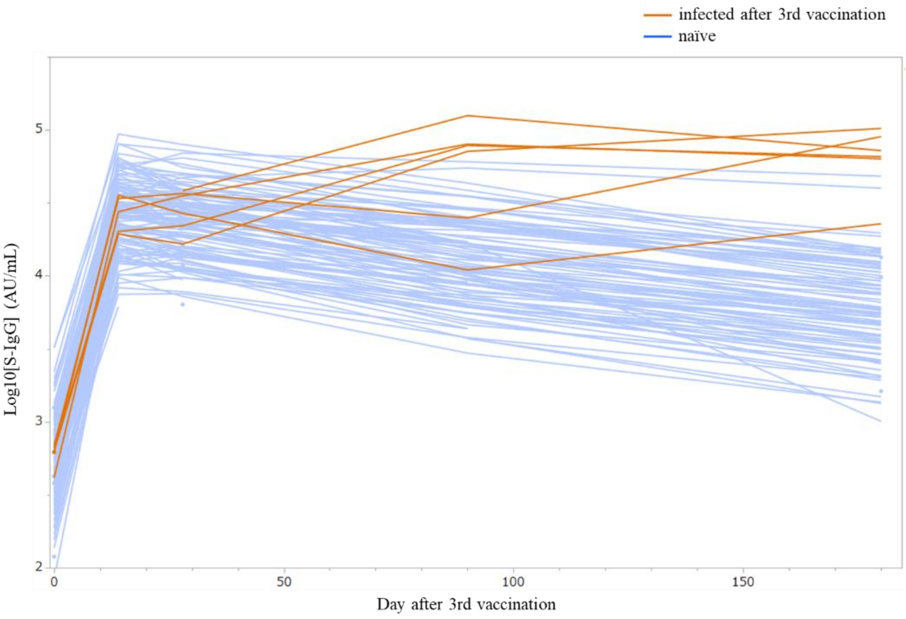

Table S1. Geometric mean titers (GMTs) of anti–SARS-CoV-2 spike protein receptor-binding domain antibodies at each measurement point by sex and age group in the naïve group.

		Day 0		Day 14		Day 28		Day 60		Day 90		Day 180		Day 270		Day 284		Day 298		Day 360		Day 450	
		n	GMT(95% CI)	n	GMT(95% CI)	n	GMT(95% CI)	n	GMT(95% CI)	n	GMT(95% CI)	n	GMT(95% CI)	n	GMT(95% CI)	n	GMT(95% CI)	n	GMT(95% CI)	n	GMT(95% CI)	n	GMT(95% CI)
Sex	Male	96	2.0 (1.7–2.4)	94	477 (395–575)	95	15,434 (12,906–18,457)	91	7526 (6578–8611)	87	3787 (3291–4357)	89	1051 (916–1205)	77	514 (448–590)	72	24,060 (20,942–27,642)	67	22,161 (19,236–25,532)	57	12,402 (10,263–14,988)	52	6339 (5068–7928)
	Female	75	1.9 (1.4–2.4)	74	597 (443–805)	70	11,621 (7819–17,270)	71	10,753 (9352–12,364)	65	5380 (4547–6365)	64	1430 (1223–1671)	62	665 (569–777)	61	27,787 (23,971–32,211)	55	23,602 (20,215–27,555)	45	12,644 (10,707–14,931)	42	5506 (4477–6772)
Age group	20–29 years	22	1.7 (1.0–2.8)	20	860 (524–1411)	20	12,817 (6815–24,102)	19	11,730 (8763–15,701)	17	5741 (3980–8280)	16	1568 (1045–2352)	14	718 (460–1119)	12	33,203 (24,171–45,609)	11	26,458 (16,934–41,338)	9	15,446 (9314–25,614)	9	6849 (3847–12,194)
	30–39 years	57	2.1 (1.6–2.8)	57	717 (573–896)	54	16,331 (12,259–21,755)	56	9055 (7682–10,673)	50	4625 (3829–5587)	52	1209 (1026–1425)	47	585 (507–676)	45	27,477 (23,168–32,587)	42	23,973 (20,443–28,112)	34	12,122 (9608–15,294)	28	6051 (4560–8029)
	40–49 years	64	1.8 (1.4–2.3)	63	473 (387–578)	64	14,300 (11,360–18,002)	61	7930 (6683–9410)	59	3989 (3324–4787)	61	1135 (957–1346)	55	560 (468–670)	52	23,256 (19,597–27,598)	46	21,823 (18,089–26,327)	39	13,168 (10,612–16,339)	37	6460 (4966–8403)
	50–59 years	23	2.0 (1.4–2.9)	23	377 (256–557)	23	12,679 (6982–23,024)	23	8796 (6539–11,831)	23	4321 (3190-5854)	22	1085 (794–1483)	21	507 (371–691)	22	24,626 (18,903–32,081)	21	20,889 (16,276–26,810)	18	10,597 (8131–13,810)	18	4444 (3265–6048)
	≥60 years*	3	1.3–3.3	3	57–759	3	5497–18,072	3	5167–8242	3	2309–4165	2	1395–1427	2	714–876	2	19,746–35,835	2	15,579–37,134	2	6210–29,276	2	3151–18,691

The range in parentheses represents the 95% confidence interval (95% CI).

* Given the small number of eligible participants, the range of antibody titers (minimum–maximum) was presented
